# The challenges of emerging HISs in bridging the communication gaps among physicians and nurses in China: an interview study

**DOI:** 10.1186/s12911-017-0473-x

**Published:** 2017-06-12

**Authors:** Dong Wen, Xingting Zhang, Jie Wan, Jing Fu, Jianbo Lei

**Affiliations:** 1 Peking University Third Hospital, 49 North Garden, Rd., Haidian District, 100191 Beijing, China; 2 Center for Medical Informatics, Peking University, 38 Xueyuan Rd., Haidian District, 100191 Beijing, China; 3 School of Nursing, Southwest Medical University, 319 Zhongshan Rd, Luzhou City, Sichuan Province 646000 China; 4 School of Medical Informatics and Engineering, Southwest Medical University, 319 Zhongshan Rd, 646000 Luzhou City, Sichuan Province China

**Keywords:** Physician-nurse communication, Information needs, Communication models, Hospital communication systems

## Abstract

**Background:**

To explore the current situation, existing problems and possible causes of said problems with regards to physician-nurse communication under an environment of increasingly widespread usage of Hospital Information Systems and to seek out new potential strategies in information technology to improve physician-nurse communication.

**Methods:**

Semi-structured interviews were conducted with 20 physicians and nurses in five leading tertiary grade A hospitals in Beijing, China (two physicians and two nurses in each hospital). The interviews primarily included three aspects comprising the current situation and problems of clinical physician-nurse communication, the application and problems of Hospital Information Systems, and assessments on the improvement of physician-nurse communication through the usage of information technology. The inductive conventional content analysis approach was employed.

**Results:**

(1) Physicians and nurses are generally quite satisfied with the current situation of communication. However, the information needs of nurses are prone to being overlooked, and the communication methods are primarily synchronous communication such as face-to-face and phone communication. (2) Hospital Information Systems are gradually being used for physician-nurse communication; in the meantime, physicians and nurses face challenges with regards to the improvement of physician-nurse communication through the usage of information technology. Challenges differ based on the different stages of using the system and the different levels of understanding of physicians and nurses towards information technology. Their dissatisfaction mainly deals with system errors and the level of convenience in using the system. (3) In-depth interviews found that in general, physicians and nurses have a strong interest and trust in improving physician-nurse communication through appropriate information technology, e.g., communication methods such as information reminders for physicians and nurses through mobile devices and instant voice-to-text conversion methods.

**Conclusions:**

There are objective risks in physician-nurse communication in Chinese hospitals, and clinical information systems lack solutions to the relevant problems. Developing a dedicated, mobile, quick and convenient module for physician-nurse communication within existing hospital information system with automatic reminders for important information that segregates between synchronous and asynchronous communication according to the different types of information could help improve physician-nurse communication.

## Background

The health challenges of China’s population are gradually emerging, and there is a serious shortage of physicians and nurses. The workload and work pressures of physicians and nurses in China are the highest in the world. As the world’s most populous country, China’s total population as of the end of 2014 amounted to 1.368 billion according to the National Bureau of Statistics of China. Of this number, those aged 65 and older amounted to 137.55 million, accounting for over 10% of the total population [1]. According to the standards of the United Nations, China is entering into a situation of population ageing, and as a result of various factors such as family planning, this trend is expected to intensify. This situation will be accompanied by rapidly increasing incidence and mortality rates for non-communicable chronic diseases among China’s population [2]. On another note, there remains a relative shortage of China’s healthcare service personnel. At the end of 2014, there were 2.89 million physicians in China (according to the 2015 China Statistical Yearbook of Health and Family Planning), translating to a physician density of 2.1 per 1,000 population. Based on average data from the Organization for Economic Co-operation and Development (OECD) countries in 2014, OECD countries had an average physician density of 3.3 per 1,000 population. On another note, the United States had a physician density of 2.6 per 1,000 population. There is also a remarkable shortage of nurses in China, with a total of 3 million nurses, translating to a nurse density of 2.2 per 1,000 population. Meanwhile, the average nurse density for OECD countries was 8.9 per 1,000 population, and the average for the United States was 11.2 per 1,000 population. The nurse-physician ratio in China was 1.04, whereas the average for OECD countries was 2.7 and the United States had an average of 4.3 nurses per physician [3]. These data demonstrate that China is facing a very serious shortage of physicians and nurses compared to the key countries in the world, and with the sustained growth of hospital patient numbers, the work pressure of medical personnel is a serious problem. A one-time nationwide survey conducted in 2016 reported that 50.8% of physicians and nurses found that the workload was overly heavy. Of those survey participants, 64.5% were physicians, and 62.9% of those physicians worked for more than 10 h per day. Likewise, only 33.6% of physicians and nurses reported that they had sufficient sleep [4].

There is a need for large volumes of information in physician-nurse communication, and good physician-nurse partnerships will help to improve physician-nurse work efficiency and relieve work pressure-related risks. Tremendous work pressures for physicians and nurses might lead to work fatigue and hence affect the consistency and quality of medical treatment. In turn, this situation leads to safety risks for patients [5, 6]. The mutual exchange of information among physicians and nurses is very important for their respective tasks, and good physician-nurse communication will help to confirm patient information and reduce clinical errors [7]. The most common information needs for physicians and nurses in clinical work are the patient’s diagnosis, drugs, and treatment, and the most common way to source out such information is through asking their colleagues [8]. The differences in the tasks handled lead to differences in information understood by physicians and nurses, and there are discrepancies in the assessments of patient conditions such as their rehabilitation and feelings [9]. During situations such as performing rounds and shift handovers, there are occurrences in which the information needs of physicians and nurses on patient conditions or follow-up status are overlooked [10]. Moreover, the physician-nurse communication model is work scene-centric, and group objectives are usually overlooked [11]. These problems will affect clinical safety. The frequency of information needs by physicians and nurses in clinical work is rather high. Cogdill et al. found that the frequency of information needs of nurses on a weekly average amounted to 0.86 times per patient [12]. Correspondingly, studies by Covel et al. found that the frequency of information needs of physicians on a weekly average amounted to 0.67 times per patient [13]. These data show that the communication of information among physicians and nurses in clinical work should be a point of focus.

The Hospital Information Systems (HISs) used widely on a global scale in recent years have led to an increased reliance of physicians and nurses towards information systems, and this scenario has given rise to opportunities and challenges for traditional physician-nurse communication models. Chinese hospitals are already widely using various types of information systems to assist them in their work. According to a report by the China Hospital Information Management Association (CHIMA), 75.26% of hospitals have implemented resident nurse work systems, 74.21% have implemented resident physician work systems, 71.05% have implemented work systems for outpatient and emergency physician stations, and 71.05% have implemented Electronic Medical Record Systems (EMRS) as of the end of 2014 [14]. HISs expedite the transmission of patient treatment information among physicians and nurses and notify specialists about consultations and decision-making advice in a quicker and better manner, resulting in higher efficiency for processes such as discharge and handover processes [15–17]. Hence, these information systems serve as an important medium of communication for physicians and nurses, allowing for a quicker understanding of the information obtained from one another while enabling convenient archiving of clinical records that facilitate long-term traceability [18]. Integrated HISs have the potential to improve the results of information communication among physicians and nurses [19], and therefore might contribute substantively in terms of generating value for China’s medical system, which has an immense service volume.

The continuous emergence of new technologies has changed traditional physician-nurse communication methods and brought about new challenges and opportunities. Currently, there is various communication methods for clinical work, and these methods can be broadly categorized into two types of communication, namely, synchronous and asynchronous [20]. Synchronous communication is the most common communication method, such as face-to-face shift handovers and phone communication, and its strength lies in a highly efficient communication rate [21]. However, the weaknesses of this form of communication include limited communication opportunities and the fact that it is prone to causing work interruptions. Asynchronous communication has thrived with the development of communication technologies such as short message service (SMS) and email technologies, and its strengths include its non-interruptive features to the work of medical personnel and its benefits in reducing medical errors [20, 22]. Additionally, the communicated contents of emails can be archived and analyzed, and data such as the frequency of communication can serve as management indicators [23].

In consideration of the constraints of the number of physicians and nurses on a global scale—particularly in China, where the problem is more serious—exploring new information needs and solutions under an environment in which physicians and nurses increasingly rely on information systems will be an important study direction. Physicians and nurses of many leading hospitals in China were interviewed for this study with the objective of exploring the opinions of physicians and opinions on physician-nurse communication and their needs relating to communication methods. There was a special focus on the effects of new information technologies on current and future communication of information among physicians and nurses and on the seeking out of new potential strategies in information technology to improve physician-nurse communication.

## Methods

### Study design

For this study, semi-structured interviews were conducted with 20 physicians and nurses in five leading tertiary grade A hospitals in Beijing, China. One of the objectives of this study was to conduct interviews to unearth further information on the issues in the questionnaire survey study regarding physician-nurse information needs that had already been published by the research group to make up for the inadequacies of the questionnaire study [24]. In that study we learned that the degree of information demand was high while degree of information satisfaction was general during the interactions between Chinese physicians and nurses and they both expected high on the inclusion of new information technologies. The interview outline was formulated based on the findings of the questionnaire study, detailed questions of studies on the communication of information among physicians and nurses based on further readings, and the experiences of researchers in terms of clinical physician-nurse communication. As shown in Table 1 below, the interviews included three aspects comprising the current situation and problems of clinical physician-nurse communication, the application and problems of information technologies, and evaluation on the improvement of physician-nurse communication through the usage of information technology.

**Table 1:** Outline of semi-structured interviews

1. Current situation and problems of clinical physician-nurse communication	What aspects of information do you communicate with nurses/physicians?
	What is the primary method of physician-nurse communication?
	Do you think that there are any communication gaps or problems?
	What type of information do you think nurses/physicians need to obtain from you?
2. Application of information technologies	What type of information systems are you using for your current work tasks?
	What are your dissatisfactions in the usage of these information systems?
	What additional needs do you have with regards to the existing information systems?
3. The possibilities of improving physician-nurse communication via information technologies	What type of effects do you feel information systems will have on physician-nurse communication?
	Which aspects require interaction with nurses/physicians through the information systems?
	Consider whether communication can be improved through information technologies.

### Study target

The interviewees were from five large-scale tertiary grade A hospitals in Beijing, China with high information technology standards. Please refer to Table 2 for the detailed information of these five hospitals [25–29].

**Table 2:** Basic information on hospitals of the interviewees

Hospital	Type	Bed numbers	Outpatient numbers	Annual number of discharged patients	Number of staff	Characteristics	Application history of information systems
Peking Union Medical College Hospital	Tertiary grade A general hospital	Over 2,000	Highest number in a single day: 15,000 persons	Over 80,000 persons	Over 4,000	National Health and Family Planning Commission (NHFPC)-appointed national referral center offering diagnostic and therapeutic care of complex and acute disorders	Its information system was established in 1981, and its online HIS was established in 1997 and has passed the interoperability maturity tests run by the NHFPC.
Peking University First Hospital	Tertiary grade A general hospital	Over 1,600	Over 2.7 million persons/year	Over 72,000 persons	Over 3,200	The first state-owned hospital in China	Its information system was established in 1993, and its inpatient and outpatient information systems were consolidated in 1995.
Peking University People’s Hospital	Tertiary grade A general hospital	Over 1,700	1.62 million persons/year	Over 43,000 persons	2,379	First general hospital built on funds raised solely by the Chinese government	The hospital launched the nation’s first comprehensive large-scale HIS pilot project in 1995, and it is the first hospital to be awarded with an HIMMS Stage 7 award in 2014.
Beijing Tian Tan Hospital	Tertiary grade A general hospital	Over 950	Over 1.4 million persons/year	Over 30,000 persons	2,787	One of the three largest neurosurgical research centers in the world	Its LAN-based HIS was established in the 1990s. Its physician-nurse work station was completed in 2001. The hospital received the Asia Pacific “Healthcare Industry” Dynamic IT award in 2006 (the only organization in China to receive the award).
Beijing Cancer Hospital	Tertiary grade A specialist hospital	Over 790	Over 450,000 persons/year	Over 40,000 persons	1,090	Domestic leader in diagnostic and therapeutic care of various common cancers in China. Internationally advanced standards.	Its information technology system was established in 2000, and its database was established in 2004.

The interviewees were representative physicians and nurses from above five hospitals. The inclusion criteria were: working in the wards, more than one years of patient care experience. The exclusion criteria were to exclude departments with less physician-nurse interaction such as outpatient, operation room and other non-clinical departments; excluding pharmacists, technicians and other non-clinical care roles; excluding interns, students and other non-resident personnel department. The job responsibilities of surveyed physicians include seeing patients, writing medical records, prescribing drugs, discussing cases, communicating with patients and interacting with nurses of treatment plan and patient progress, etc. Some physicians also undertake outpatient surgery, ward management responsibilities. The job responsibilities of surveyed nurses include receiving patients, inspecting wards, writing nursing records, auditing, processing and executing orders, or reporting the condition of patients, distributing drugs, communicating with patients, etc.

The interviewees comprised 20 physicians and nurses of these five target hospitals. Two physicians and two nurses were interviewed from each hospital, and the interviewees were recommended by contacts in each hospital. The interviews were conducted and recorded upon obtaining the approval of the interviewees, and the interview recordings do not reveal sensitive personally identifiable information such as the names and contact methods of the interviewees.

### Data analysis

Upon completion of the interviews, the interviews were transcribed from audio into text, and the interview contents were analyzed using the inductive conventional content analysis approach [30]. Researchers read the transcribed texts several times to understand the information conveyed in the interviews. The key information of the interview contents was extracted, coded, and subsequently categorized in six themes: content of communication, information needs, communication means, usage of information systems, impact of information systems and expected communication improvement strategies. Then the researchers analyzed and summarized the common and unique opinions of each theme in the same role, and finally compared the discrepancies between the two roles of nurses and physicians. At the end all co-authors reviewed the transcribed encodings and the collected viewpoints of the interviews, and finally developed a consensus of the content analysis after further discussion and adjustment.

## Results

### Basic information of the interviewees

The basic information of the interviewees for this study is shown below in Table 3. The interviewees comprised 10 physicians and 10 nurses; two physicians and two nurses were interviewed from each hospital. The interviewees comprised 6 males and 14 females mainly aged between 30–40 years, and their work locations were mainly in the wards. The interviewee physicians held the roles of resident physician, attending physician and chief physician, and they all held master’s and PhD qualifications. The interviewee nurses held the roles of nurse, senior nurse and supervisor nurse, and they all held bachelor’s or lower qualifications.

**Table 3:** Basic information of the interviewees

Item	Selected options	Number of persons
Gender	Male	6
	Female	14
Age	20–30 years old	5
	30–40 years old	11
	40–50 years old	4
Educational level	PhD	9
	Master’s degree	1
	Bachelor’s degree	5
	Below Bachelor’s degree	5
Role	Chief physician	2
	Attending physician	6
	Resident physician	2
	Supervisor nurse	4
	Senior nurse	4
	Nurse	2
Place of work	Ward	14
	Outpatient and ward	6

### Current situation of physician-nurse communication

The physicians and nurses were generally quite satisfied with the current situation of clinical communication. First, nearly all of the interviewees mentioned that there were no obvious problems in terms of physician-nurse communication, and the main reasons were that the job responsibilities for clinical work are quite clear and that there have not been obvious communication barriers due to long-term cooperation. Notably, some interviewees indicated certain factors that affected communication in their actual work processes, including communication efficiency being related to personal communication skills, not being able to explain matters in detail when one is busy with work, and having to wait for responses at times. However, these problems can all be solved through further communication, e.g., through measures such as communication skills training, further communication during breaks, and narrowing the distances between offices.

*When asked about if there are any gaps or problems in physician-nurse communication, one physician answered, “I don’t think there’s any problem. Sometimes, the work pressure of the nurses is relatively large and hard, and sometimes there is not enough time to explain or say anything. If I couldn’t get the right answers I will try to ask other physicians if they know.”*

However, there are objective inadequacies in terms of physician-nurse communication, and the information needs of nurses are prone to being overlooked. First, the information needs of physicians and nurses differ. The information needs required by physicians from nurses mainly include manifestations of the patient’s illness, complaints of discomfort, and prescription checking, such as vital signs, adverse reactions, and rules of issuance for e-prescriptions for new physicians to learn. The information needs required by nurses from physicians mainly include treatment program updates and things to take note of, such as new drug usage methods, test and examination results, and confirmation of prescriptions. Second, with regards to the understanding shown by physicians and nurses on the information needs of the other party, nearly all nurses showed a rather consistent level of understanding towards the information needs of physicians, and their understandings tally with the actual needs of physicians. However, physicians need more information from nurses than the other way round, nurses less frequently mentioned certain information needs required by physicians of nurses such as nursing assessments and how to prevent patients from falling, showing that the information needs of physicians from nurses is higher than what was predicted by nurses. A part of nursing information provided by nurses is very important to the physicians’ work, however the nurses may not realize the importance of that information, and the phenomenon might imply that the recognition of self-worth of nurses in clinical partnerships is lower than what it should be. However, different physicians showed rather considerable discrepancies in expressing the information needs required by nurses. Different physicians’ understanding of nurses’ information needs is not consistent and not comprehensive. The understanding shown by physicians covered the information needs of nurses and was basically consistent with the information needs of nurses overall, and this fact confirms the conclusion that physician-nurse partnerships are in a good state. However, the feedback provided by different physicians revealed different aspects of understanding, and this result shows that individual physicians do not have a comprehensive understanding of the information needs of nurses. If the physicians do not have an accurate and comprehensive judgment on the information needs of nurses in clinical work, there may be a part of the information the physician will not take the initiative to inform the nurse, the nurses then have to ask to obtain this information; otherwise the information needs of nurses might be overlooked.

*When asked about what information that nurses needed from physicians, one physician replied as: “on the one hand, the current situations of patients since nurses need to know this by themselves; on the other hand, the next treatment plan since nurses might be asked about this by patients frequently since they interact with patients more than physician with patients.”*

*Another physician answered the same question as: “Since the nurses were graduated from nursing profession, they might not know what exactly an order is for except for some commonly used drugs. It is highly possible that patients may ask about this when they administer drugs to patients, therefore it is helpful to nurses if they have general understanding about the mechanism of drugs by asking physician beforehand.”*

Currently, physician-nurse communication is primarily conducted through synchronous communication methods such as face-to-face and phone communication, whereas HISs are also used for the sharing of information. All physicians and nurses from different hospitals mentioned that the current primary method of communication is through phone and face-to-face communication, and they expressed a higher level of approval for phone communication as they feel that this form of communication allows for the quick solving of problems. However, they are more inclined to face-to-face communication for urgent and important matters. For the issue of phone communication resulting in interruptions at work, studies by Coiera et al. [20] found a different conclusion on whether synchronous communication methods pose major interruptions to clinical work in a hospital environment. Interviewees found that the time schedules for clinical tasks are not stringent, and interruptions at work caused by phone calls are acceptable at regular occurrences.

*When asked about whether the synchronous communication may interrupt workflow, one physician answered: “Because our work itself is not exactly planned, much of our work happened all of a sudden or temporarily, so it is acceptable to be interrupted.”*

Conversely, in terms of asynchronous communication, nearly all interviewees mentioned that physicians would utilize nursing records of patients through information systems and that nurses would utilize medical records by physicians through information systems.

*When asked about the communication means between physicians and nurses, one nurse answered: “Using the information system reduces the dependency of physicians for information since we can check the information systems for information we might need to know.”*

This fact indicates that although information systems play an important role in physician-nurse communication, the systems do not completely cover all issues requiring communication among physicians and nurses. Additionally, communication methods such as We Chat messaging, SMS messaging, and emails have also been used in hospitals, and such methods are mainly used when one is out-of-office or for public announcements. However, the interviewees also said that such communication methods might result in delayed transmission of information and thus delay decision-making processes.

### Usage of information systems

Physicians and nurses use information systems frequently in their work, and they can adequately use the system once they become accustomed to it. The interviewees mentioned that the main information systems used by physicians in their work are electronic medical records, prescription systems, laboratory examination systems, and imaging systems, and they use tablets to check medical records when doing rounds. In addition, the main information systems used by nurses in their work are prescription systems, electronic medical records, and nursing information systems. Nurses use prescription systems to check prescriptions, electronic medical records or mobile nursing systems to record vital signs and nursing records, and mobile nursing systems to implement prescriptions. On another note, some interviewees with administrative roles mentioned that Office Automation (OA) systems are also used for processes such as email processing and receiving notifications. With regards to the usage of current HISs, all of the interviewees said that they were not accustomed to the HIS when they first started using it, and there were several problems over the process of using the information systems, but they were able to become accustomed to the systems upon long-term usage.

*When asked about what would be the problems by using information systems, one physician answered: “At the beginning of using the system, there were more problems since we were all not familiar with the information system, but after one period of getting used to it there is no that many problems anymore.”*

The physicians and nurses raised many suggestions regarding the improvement of clinical information systems, and their dissatisfaction mainly regarded system errors and the level of convenience in using the system. For example, most of the interviewees complained that the actual functions might cause inconvenient usage such as complex functions and processes, unclear display of medical records, slow access to the contents of medical records, the lack of a retrieve function for electronic medical records, and inconvenient keying in of medical records. There was also mention of an even more serious situation whereby the unclear directions in the prescription system led to the issuance of erroneous prescriptions; although this type of error was not due to drug usage errors by the physicians, it caused additional losses in work efficiency. Additionally, the interviewees noted that physicians and nurses use different information systems, and the information is not consistent across most systems, with some information not being shareable. On another note, the interviewees mentioned that they have encountered occasional system errors such as network disruptions and software crashes that significantly affected their work efficiency and regular operations; these situations even led to a loss of information, and prompt servicing by the manufacturer of HISs is required to fix the problem.

### How information technologies assist physician-nurse communication

Currently, clinical information systems assist physician-nurse communication, and improved information technology methods and the usage of appropriate information technologies are expected to improve physician-nurse communication. Physicians and nurses generally admitted that they will proactively determine the clinical information of the other party through information systems, and new technologies can improve work efficiency, such as voice-to-text conversion technologies, which can help expedite the writing of medical records. However, with regards to whether information systems can improve physician-nurse communication, there are two types of opinions. Some interviewees believe that information systems cannot improve physician-nurse communication mainly because clinical work is hectic and it is not possible to always look for computer notifications and key in records at any given time. Furthermore, this group believes that the feedback provided through information systems is delayed and can cause work delays.

Conversely, some interviewees feel that information systems can improve physician-nurse communication because they can currently determine a significant amount of necessary information via information systems. Upon discussing different new information technologies among the interviewer and interviewees, the interviewees generally showed that they are very supportive and expectant of the usage of appropriate information technologies in improving physician-nurse communication. Some of the appropriate technologies include a body-worn voice recorder to record working notes to ensure better understanding and implementation of the information transmitted and a body-worn mobile pager to obtain notifications at any time to ensure smoother communication. Other appropriate technologies include message notifications through a mobile app available any time, which piggybacks on the current usage of tablets when conducting rounds.

*When asked about what more expectations that they want, both physicians and nurses expressed that sometimes it is inconvenient to access patient information from the current hospital information systems, sometimes they were occupied by too much information. It might be helpful to offer just needed information anywhere and anytime when there is a need, mobile access and intelligent information push to mobile phone terminal will be highly expected.*

## Discussion

This study is an important supplementary study to a previously conducted related questionnaire survey, and more valuable information can be found throughout this study. The previous quantitative questionnaire findings [24] showed that physicians and nurses have high information needs in terms of their interaction with one another, but their needs are only fulfilled in a mediocre manner. Physicians have rather high expectations for communication and information technologies, and a semi-structured interview could help reveal more information. First, the survey respondents for the questionnaire survey might have misinterpreted the questions or available options, and interviews could help to provide a more accurate and detailed understanding of the actual thoughts of the interviewees and unearth more information. Furthermore, via the process of guiding the interviewees through the analysis of a question, interviewers may understand a complete thought that interviewees haven’t formed. Second, in consideration that physicians and nurses might be used to the current work methods or lack understanding towards information technologies, the interview methods employed interactive discussions to stimulate the thought processes of the interviewees, and examples and statements were used to discover the level of interest and acceptance of the interviewees towards new technologies. Third, the study compared the extent to which the responses matched by conducting separate peer interviews with physicians and nurses and, through this approach, derived a more objective conclusion by determining the communication gaps among physicians and nurses.

There may be bias regards the dissatisfaction of using the information systems and additional requirements in this semi-structured interviews. As clinical staff and information system users, respondents may lack familiarity with the information system or they provide unreasonable demand. However, in the process of analysis, this conclusion come from the collection and summary of these two aspects showing that these viewpoints may be common and has not much connection with personal experiences. In addition, as trained clinical informatician, the researchers have both in-depth understanding of the hospital information system and clinical experience, they can fully understand the answers from the respondents therefore the analysis of the interview results should be relatively consistent with the clinical work of real environment and in accordance with the logic of hospital information systems.

This study was launched by five well-known leading hospitals in China comprising general hospitals and a specialist hospital. The differences in the actual work models, adopted information systems, and hospital environments of the different hospitals were instrumental in aiding the understanding of diversified and different types of information as these scenarios are representative of the general situations of medical personnel in mainstream tertiary grade A hospitals in China.

In terms of communication methods, physicians and nurses are accustomed to synchronous communication methods, and communication efficiency could be improved through the usage of asynchronous communication with appropriate information technologies [22]. Chinese hospitals tend to use synchronous communication methods more frequently because their work is intense and failure to provide prompt responses will cause work delays. However, in actual practice, although urgent matters must be communicated via synchronous communication, asynchronous communication is more appropriate for memos. The transmission of memos such as administrative notices and patient check-up reminders is of high priority but not urgent. The key action for memos is that the recipient must provide acknowledgment of receipt so that the sender of the message can confirm that the relevant message has been received. Over-reliance on synchronous communication for unimportant information might result in misplaced priorities in clinical work tasks and in turn lead to reduced productivity. Thus, asynchronous communication should be used in clinical work, whereby appropriate information technologies are used, and special physician-nurse communication platforms are established through the analysis of practical clinical information needs of physicians and nurses. Methods such as mobile terminals, instant messaging, discussion groups, retention of read receipts, and reading logs can be employed, and information that is of a lower priority that can be communicated through asynchronous communication in clinical work can be segregated to prevent scenarios whereby physicians and nurses must process too much information during their limited, tight clinical working hours.

Although most of the interviewees expressed that they are satisfied with physician-nurse communication and are accustomed to the usage of information systems, further interviews found that physicians and nurses face different challenges in using information systems at different stages, and the problems faced and issues focused upon differ from person to person. Hence, if the sentiments of physicians and nurses were not unearthed in depth, they cannot reflect the current situation and expectations of physician-nurse communication. First, the interviewees noted that there were several problems when first using the new information systems, and this fact shows that a substantial amount of learning is required for physicians and nurses to master new information technology systems. Additionally, operational errors might be committed due to unfamiliarity with information systems; therefore, physicians and nurses are quite cautious when using new communication methods. Menachemi et al. also noted that although electronic medical records have many benefits, there might be a loss in productivity due to work disruptions while learning to use the new systems [31]. Second, during the period of becoming accustomed to using the information systems, the interviewees tend to become passive and reliant on information systems. Furthermore, because physicians and nurses are not experts on information technology and the nature of their work is rather hectic, they will not have the time to fully and systematically consider in-depth whether there are problems in physician-nurse communication. Hence, the interviewees generally concluded that they are rather satisfied with physician-nurse communication. The inclination towards developing a reliance on information systems is a scenario that many hospitals face when their information systems have reached a certain stage since the establishment of these systems. Third, for experienced physicians and nurses using these information systems for a long time, or upon informing and making known to the interviewees of the development and research updates of information technologies through the interviewer, we found that the interviewees generally had a deeper understanding of the current situation of physician-nurse communication (more problems were discovered) and that they had very high levels of expectations and approval for new information technologies. On the one hand, physicians and nurses who are experienced and savvy with information technologies will proactively use information systems to look up patient information, but the clinical information required by physicians and nurses are separately located in different systems. It is inconvenient to retrieve the necessary information promptly through information systems, and the levels of importance and priority are not clearly shown when different portions of information are presented to physicians and nurses. On the other hand, clinical physicians and nurses have already began to use personal digital assistants (PDAs) or tablets to look up information or make records, but mobile devices have not been built with consideration for physician-nurse communication. Should medical personnel be physically away from phones and computers, they might not or will not be able to retrieve patients’ treatment information promptly and will not be able to communicate with colleagues in a prompt manner, going beyond the outreach of the collaborative team. Hence, using mobile terminals such as mobile phones that are more portable and commonly used will allow for intelligent, automatic, and user-selectable information notifications for physicians and nurses so that they can maintain a state of cooperation while not being bombarded with too much information [32]. Furthermore, the current primary mode of clinical physician-nurse communication in China is still face-to-face and phone communication. Although there are many types of information systems, there is no platform dedicated to physician-nurse communication, and none of the systems fully demonstrate the advantages of the aforementioned types of information technologies.

In consideration of the existing limitations of physician-nurse communication mentioned above, it is worthwhile to conduct discussions and research & development on how to establish a more convenient and efficient physician-nurse communication model. Currently, clinical information systems mainly include e-prescription systems, EMRS, test and examination systems, and nursing information systems. Most of these systems are geared towards certain roles and functions in terms of usage, and no system has been established to facilitate close physician-nurse communication and information sharing. The current clinical information systems are not all tied to the most commonly required types of information in physician-nurse communication such as confirmation of prescription changes, real-time feedback on patient conditions, and queries on drug effects. With regards to the existing physician-nurse communication risks discovered in this study, we are of the opinion that it is necessary to establish a mobile and convenient physician-nurse communication module that is dedicated to communication purposes, provides automatic notifications of important information, and segregates between synchronous and asynchronous communication according to the different types of information. The suggestions for improvement to the existing physician-nurse communication models raised by the interviewees are very good points to take heed of. These suggestions include improving the way in which information is presented in HISs, providing information reminders through pagers or mobile apps, and realizing instant voice-to-text conversion of information. Connecting physician-nurse communication through smart phones might help to integrate synchronous and asynchronous communication perfectly [33]. Urgent queries in clinical work could be communicated directly through voice calls, while reply reminders can be sent through notifications for clinical matters that can be handled later. Office matters or standard reminders could be disseminated through notice boards and other methods, and the relevant personnel could be notified to check the notices.

Currently, physician-nurse communication in Chinese hospitals is primarily conducted through synchronous communication methods, and there are objective risks in physician-nurse communication in the clinical environment. The levels of understanding towards the communication of information among physicians and nurses differ based on the different stages that physicians and nurses are at in using the information systems and their experiences thereof. They are highly expectant of the improvements to physician-nurse communication through the usage of appropriate new information technologies. There has yet to be a good dedicated solution for clinical information systems to improve physician-nurse communication, and the reasonable usage of information technologies is expected to improve the current situation. Hence, it is highly meaningful to develop a mobile and convenient physician-nurse communication module integrated into the existing information flow that is dedicated to physician-nurse communication, provides automatic notifications of important information, and segregates between synchronous and asynchronous communication according to the different types of information.

## Conclusion

Currently, physician-nurse communication is primarily conducted through synchronous communication methods in Chinese hospitals. There are objective risks in physician-nurse communication in clinical settings. Perceptions of physicians and nurses differ based on different stages and roles of using HISs and different experiences. Physicians and nurses have strong expected in improving physician-nurse communication through appropriate information technology. Developing a dedicated, mobile, quick and convenient module based on existing hospital information system for physician-nurse communication with automatic reminders for important information that segregates between synchronous and asynchronous communication according to the different types of information is significant.

## Abbreviations

CHIMA: China hospital information management association
EMRS: Electronic medical record systems
HISs: Hospital information systems
NHFPC: National health and family planning commission
OA: Office automation
OECD: Organization for economic co-operation and development
PDAs: Personal digital assistants
SMS: Short message service
